# Safety of integrated preventive chemotherapy for neglected tropical diseases

**DOI:** 10.1371/journal.pntd.0010700

**Published:** 2022-09-29

**Authors:** Allan M. Ciciriello, Jessica K. Fairley, Emma Cooke, Paul M. Emerson, Pamela J. Hooper, Birgit Bolton, Genevieve LaCon, David G. Addiss

**Affiliations:** 1 International Trachoma Initiative, The Task Force for Global Health, Decatur, Georgia, United States of America; 2 Division of Infectious Diseases, Department of Medicine, Emory University School of Medicine, Atlanta, Georgia, United States of America; 3 Departments of Medicine and Pediatrics, Baylor College of Medicine, Houston, Texas, United States of America; 4 Public Health Informatics Institute, The Task Force for Global Health, Decatur, Georgia, United States of America; 5 Focus Area for Compassion and Ethics, The Task Force for Global Health, Decatur, Georgia, United States of America; Beijing Children’s Hospital Capital Medical University, CHINA

## Abstract

**Background:**

Preventive chemotherapy (PC) is a central strategy for control and elimination of neglected tropical diseases (NTDs). Increased emphasis has been given to “integration” of NTD programs within health systems and coadministration of NTD drugs offers significant programmatic benefits. Guidance from the World Health Organization (WHO) reflects current evidence for safe drug coadministration and highlights measures to prevent choking of young children during PC.

**Methodology:**

To understand how coadministration of NTD drugs might affect PC safety, we reviewed literature on choking risk in young children and safety of coadministered NTD drugs. To understand current practices of drug coadministration, we surveyed 15 NTD program managers and implementing partners.

**Principal findings:**

In high-income countries, choking on medication is an infrequent cause of death in young children. In low-resource settings, data are limited, but age-appropriate drug formulations are less available. During PC, fatal choking, although infrequent, occurs primarily in young children; forcing them to swallow tablets appears to be the major risk factor. The WHO currently recommends 6 drugs and 5 possible drug combinations for use in PC. Of 105 nations endemic for the 5 PC-NTDs, 72 (68.6%) are co-endemic for 2 or more diseases and could benefit from drug coadministration during PC. All 15 survey respondents reported coadministering medications during PC. Reported responses to a child refusing to take medicine included: not forcing the child to do so (60.0%), encouraging the child (46.7%), bringing the child back later (26.7%), offering powder for oral suspension (POS) for azithromycin (13.3%), and having parents or community members intervene to calm the child (6.7%).

**Conclusions:**

Coadministration of NTD drugs during PC appears to be increasingly common. Safety of coadministered PC drugs requires attention to choking prevention, use of approved drug combinations, and increased access to age-appropriate drug formulations.

## Introduction

Periodic treatment of at-risk populations, known as preventive chemotherapy (PC), is a central strategy for control and elimination of neglected tropical diseases (NTDs), including trachoma, onchocerciasis, lymphatic filariasis (LF), schistosomiasis, and soil-transmitted helminthiasis (STH). Between 2016 and 2019, more than 1 billion people received PC each year, resulting in significant reductions in morbidity, reduced transmission, and for some NTDs, regional elimination as a public health problem [1]. This figure was reduced to 732 million during 2020 because of the Coronavirus Disease 2019 (COVID-19) pandemic [2]. When delivered at the population level in response to a public health prevalence threshold being exceeded, and without individual diagnosis, PC is referred to as mass drug administration (MDA); it can also be targeted to specific groups, for example, to school-age children, as is often the case for schistosomiasis and STH [3].

With the maturation of disease-specific NTD programs during the past 2 decades, increased emphasis has been given to their “integration” within health systems [4]. Momentum for integrated approaches to PC reached a turning point in 2011, when the World Health Organization (WHO) published its first NTD Roadmap calling for “all NTD-endemic countries to have a plan of action for integrated PC by 2015” [5]. The push for programmatic integration has been driven by the co-endemicity of many NTDs and the desire for improved program efficiency and lower cost [6,7]. Integration requires a high degree of coordination with logistics, training, community messaging, and management and reporting of serious adverse events (SAEs) [8,9].

Shared logistics, planning, training, and coadministration of drugs during MDA are key components of integration and offer significant cost-savings and programmatic benefits [10–12]. However, coadministration also presents potential safety concerns, including potential pharmacological interactions; increased risk of choking while swallowing multiple tablets at the same time; and the possibility of confusion or incorrect dosing when giving drugs with different formulations (e.g., tablets and oral suspension), exclusion criteria (e.g., age, pregnancy), and recommendations for safe administration (e.g., crushing some, but not all, tablets for children below a certain age) [13–16].

The WHO updated PC safety guidance in 2021, highlighting which drugs can be safely coadministered [16]. It also emphasizes practical measures to prevent choking of young children. Fatal choking during deworming school-age children was first reported by the WHO in 2007. Despite recommendations to prevent choking by crushing deworming tablets before giving them to young children and not forcing children to take NTD medicines against their will, risk of fatal choking during PC administration has not been eliminated [13,14,17,18].

## Methods

To understand how coadministration of NTD drugs might affect the risk of choking and other challenges to safety, we (1) reviewed WHO data on endemicity of the 5 NTDs currently addressed through PC; (2) searched databases for information on safety of coadministered NTD drugs; (3) reviewed published literature on choking risk in young children; and (4) surveyed 15 NTD program managers and implementing partners to gain a preliminary indication of current practices and attitudes towards drug coadministration.

The WHO’s Preventive Chemotherapy and Transmission Databank was used to determine endemicity of the 5 NTDs. Officials in the Department of Neglected Tropical Diseases at the WHO provided data on the number of persons treated with drug combinations during PC for the years 2010 to 2020. PubMed and Embase were searched for literature on the general choking risk in young children, as well as for choking and other safety hazards during PC. Google Scholar, PubMed, Cochrane, Scopus, and ClinicalTrials.gov were searched for studies of pharmacokinetics, safety observations, and randomized controlled trials of the NTD drug combinations. The search strategies can be found in the Supporting information **(S1 Appendix)**.

To gather preliminary information on safety practices in PC programs, informal surveys were conducted with a convenience sample of 15 NTD program managers and representatives of non-governmental organizations (NGOs) that partner with the International Trachoma Initiative. All respondents were engaged in MDA for trachoma, as well as for other NTDs (median of 5 NTDs per respondent).

Interviews were conducted in English, French, or Spanish between March 6th and May 19th, 2020, through video conferencing (3 respondents) or email (12 respondents for whom video conferencing was inconvenient because of limited internet bandwidth or scheduling conflicts). The same questions were asked of all respondents (**S1 Questionnaire**). Information was collected on drug coadministration, tablet crushing, parental involvement in administering medication, child refusals, and NTD integration. Responses between different groups of respondents were tested for statistical significance using the Fisher exact test (Epi-Info, Version 7.2.5.0, CDC, Atlanta, Georgia, United States of America). The intent of these 15 interviews was not to make generalizable claims about PC safety practices, but rather, to gain an initial “snapshot” of practices across programs.

## Results

### WHO data on neglected tropical disease endemicity and recommendations for coadministration of drugs

Of 105 nations considered by WHO to be endemic for at least 1 of the 5 NTDs for which PC is recommended, 72 (68.6%) are endemic for 2 or more diseases and 14 (13.3%), mostly in sub-Saharan Africa, are endemic for all 5 NTDs (**Table 1**) [19–23]. Not all districts within these countries are co-endemic for all NTDs. For example, Burundi is endemic for schistosomiasis and STH, but only 11 (64.7%) of its 17 provinces are co-endemic for both diseases [24,25].

The WHO currently recommends 6 drugs and 5 drug combinations for use during PC. Albendazole is coadministered with either diethylcarbamazine (DEC) or ivermectin for LF, and, in some areas, all 3 drugs are coadministered for LF elimination (this combination is abbreviated as “IDA”) (**Table 2**) [26–29]. Mass treatment with IDA is also effective against onchocerciasis and STH. Praziquantel is often given with either albendazole or mebendazole, targeting both schistosomiasis and STH [26]. Use of drugs for PC, either alone or in combination, is approved by national drug regulatory agencies in each country.

**Fig 1:**
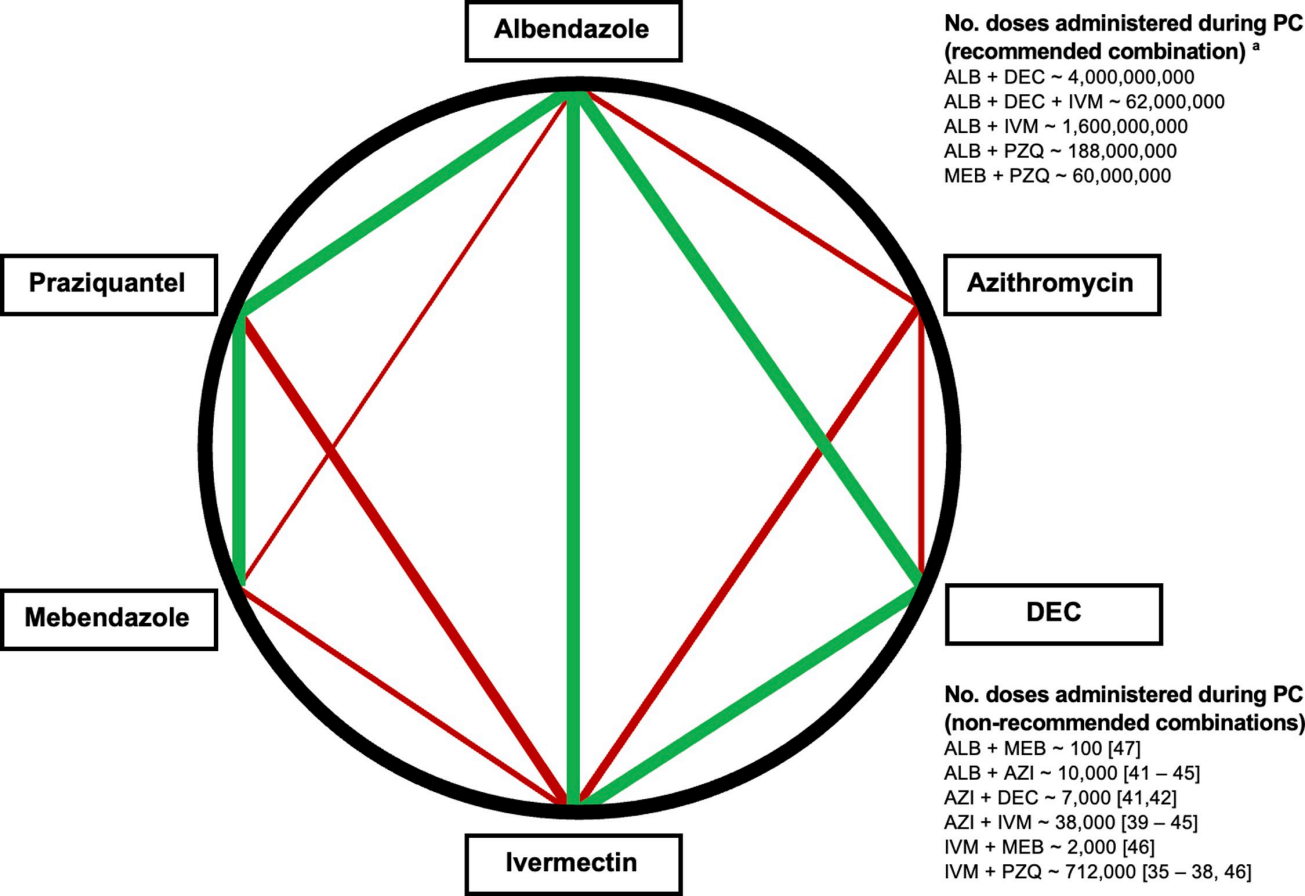
NTD drug coadministration safety Evidence strength. Green lines represent WHO-recommended combinations. Red lines represent non-WHO-recommended combinations. Thicker lines represent more available safety data. ^(a)^ Personal communication, Drs. Jonathan King and Denise Mupfasoni, WHO; data from 2010–2020. **ALB,** albendazole; **AZI,** azithromycin; **DEC,** diethylcarbamazine; **IVM,** ivermectin; **MEB,** mebendazole; **PZQ,** praziquantel.

For other patterns of NTD co-endemicity, additional drug combinations have been tested for safety and efficacy and used in pilot programs, but are not yet recommended by the WHO [35–47]. For example, the WHO does not recommend coadministration of azithromycin with other NTD drugs or coadministration of praziquantel with ivermectin or DEC. This currently limits coadministration in settings where trachoma is co-endemic with other NTDs or where schistosomiasis is co-endemic with LF or onchocerciasis. **Fig 1** illustrates the number of persons known to have received different combinations of coadministered NTD drugs, based on WHO records and the published literature. Pharmacokinetic studies reveal no significant interactions or toxicity with these drug combinations, and incidence and severity of serious adverse reactions are generally not increased by coadministration. A full list of the studies referenced in **Fig 1** can be found in **Tables 3** and **S1**.

**Table 1:** Reported co-endemicity of 5 NTDs, by country.

**NTD endemicity, by country**					
	**Disease**				
**Country**	LF	ONC	SCH	STH	TRA
Afghanistan					
Angola					
Benin					
Botswana					
*Brazil*					
Burkina Faso					
Burundi					
Cambodia					
Cameroon					
Central African Republic					
*Chad* *					
*Colombia* *					
Comoros					
Congo					
Côte d’Ivoire					
*Democratic Republic of the Congo* *					
Egypt					
Equatorial Guinea					
Eritrea					
Eswatini					
*Ethiopia* *					
Fiji					
Gabon					
Gambia					
Ghana					
*Guatemala*					
Guinea					
Guinea-Bissau					
Guyana					
*Haiti*					
India					
Indonesia					
Kenya					
Kiribati					
Lao PDR					
Liberia					
Madagascar					
Malawi					
Mali					
Mauritania					
Micronesia					
*Mozambique* *					
Myanmar					
Namibia					
Nauru					
Nepal					
*Niger* *					
*Nigeria* *					
Pakistan					
*Papua New Guinea* *					
Peru					
Philippines					
Rwanda					
Sao Tome and Principe					
*Senegal*					
Sierra Leone					
Solomon Islands					
Somalia					
South Africa					
*South Sudan* *					
Sudan					
Timor-Leste					
Togo					
Tuvalu					
*Uganda* *					
*UR Tanzania* *					
*Vanuatu* *					
Venezuela					
Viet Nam					
Yemen					
Zambia					
Zimbabwe					

Source: WHO [19–23]. Shaded cells represent disease endemicity at levels high enough to recommend PC. Italicized names represent countries in which survey respondents work (see also **Table 4**).

*Represents countries in which survey respondents reside.

LF, lymphatic filariasis; NTD, neglected tropical disease; ONC, onchocerciasis; SCH, schistosomiasis; STH, soil-transmitted helminths; TRA, trachoma.

**Table 2:** Features of drugs used in preventive chemotherapy to control and eliminate neglected tropical diseases.

Feature	Neglected Tropical Disease				
	LF	Onchocerciasis	Schistosomiasis	STH	Trachoma
	Drug Information				
Drug (Company donating drug for PC) *Formulation*	Ivermectin (Merck) *Tablet* Albendazole (GlaxoSmithKline) *Chewable tablet* DEC (Eisai) *Tablet*	Ivermectin (Merck) *Tablet*	Praziquantel (Merck KGaA) *Tablet*	Mebendazole ^a^ (Johnson & Johnson) *Chewable tablet* Albendazole ^a^ (GlaxoSmithKline) *Chewable tablet*	Azithromycin (Pfizer) *POS* ^b^ Azithromycin (Pfizer) *Tablet*
Dimensions of donated tablets	Ivermectin 1 x 6 x 6 mm Albendazole 6 x 9 x 19 mm DEC 2 x 9 x 9 mm	1 x 6 x 6 mm	6 x 8 x 22 mm	Mebendazole ^a^ 2 x 15 x 15 mm Albendazole ^a^ 6 x 9 x 19 mm	5 x 6 x 14 mm
WHO-recommended lower age for PC	2 years (DEC)	Approximately 5 years	Approximately 6 years	12 months	6 months for POS 7 years for tablets
WHO-recommended lower height for PC	90 cm (ivermectin)	90 cm	94 cm	Not based on height	120 cm for tablets
	**Preschool-Age Children-Specific Information**				
No. at-risk preschool-aged children for whom PC is recommended (millions)	Approximately 38 ^c^	Not currently recommended	Not currently recommended	299.2 [30]	Approximately 23 ^d^
No. at-risk preschool-aged children receiving PC (millions)	33.9 [31]	Not currently recommended	Not currently recommended	165.2 [30]	Approximately 5 ^e^
Estimated median trachea diameter at lower age for PC [32]	5 mm	7 mm	7 mm	4 mm	8 mm

^a^For STH, the GSK donation of albendazole and the J&J donation of mebendazole to the WHO are primarily intended for school-age children, although J&J’s rapidly-disintegrating, chewable mebendazole formulation is also donated for preschool-aged children. Generic drugs, primarily albendazole, are purchased by governments and non-governmental organizations from several manufacturers for preschool-age children. Generic tablets vary considerably in size, with albendazole commonly being 4 x 13 x 13 to 5 x 9 x 20 mm in diameter. J&J’s rapidly-disintegrating, chewable mebendazole formulation is also donated for pre-school-aged children.

^b^For trachoma, powder for oral suspension (POS) is recommended for children ≥ 6 months to < 7 years of age or anyone < 120 cm in height.

^c^Calculated by dividing reported number of preschool-aged children receiving PC in 2019 by reported coverage rate of 89.2% [31].

^d^Calculated by multiplying the number of people living in districts with trachomatous follicular inflammation of ≥ 5% in 1 – 9-year-olds in 2020 (154.5 million) by the percentage of people 0 – 4 years old in Ethiopia (14.6%) [33,34]. An estimated 49% of the global burden of trachoma is in Ethiopia [33].

^e^Calculated by multiplying the reported number of people who received antibiotics for trachoma in 2020 (32.8 million) by the percentage of people 0 – 4 years old in Ethiopia (14.6%) [33,34].

**LF:** lymphatic filariasis; **PC:** preventive chemotherapy; **POS:** powder for oral suspension; **STH:** soil-transmitted helminths.

**Table 3:** Estimated number of persons receiving coadministered drugs used in preventive chemotherapy for NTDs, by drug combination, as well as source of these estimates.

Drug combination	Total estimated no. of persons receiving combination	Sources	No. subjects studied	Location	Evidence type
Albendazole Diethylcarbamazine	4,000,000,000	WHO^a^ ^b^	N/A	Worldwide	Program
Albendazole Ivermectin	1,600,000,000	WHO^a^ ^b^	N/A	Worldwide	Program
Albendazole Praziquantel	188,000,000	WHO^a^ ^b^	N/A	Worldwide	Program
Albendazole Diethylcarbamazine Ivermectin	62,000,000	WHO^a^ ^b^	N/A	Worldwide	Program
Mebendazole Praziquantel	60,000,000	WHO^a^ ^b^	N/A	Worldwide	Program
Albendazole Ivermectin Praziquantel	710,397	Eigege A et al. [35]	5,084	Nigeria	Safety
		Mohammed KA et al. [36]	705,055	Zanzibar	Safety
		Na-Bangchang K et al. [37]	23	Thailand	PK
		Namwanje H et al. [38]	235	Uganda	RCT
Azithromycin Ivermectin	27,479	Marks M et al. [39]	1,291	Solomon Islands	RCT
		Romani L et al. [40]	26,188	Solomon Islands	Safety
Albendazole Azithromycin Diethylcarbamazine Ivermectin	7,318	John L et al. [41]	37	Papua New Guinea	PK
		John L et al. [42]	7,281	Papua New Guinea	RCT
Albendazole Azithromycin Ivermectin	3,047	Amsden GW et al. [43]	18	USA	PK
		Coulibaly YI et al. [44]	3,011	Mali	RCT
		El-Tahtawy A et al. [45]	18	USA	PK
Ivermectin Mebendazole Praziquantel	2,032	Ndyomugyenyi R et al. [46]	N/A	Uganda	Safety
Albendazole Mebendazole	110	Speich B et al. [47]	N/A	Tanzania	RCT

^a^Personal communication, Drs. Jonathan King and Denise Mupfasoni, WHO; data from 2010–2020.

^b^Supporting studies for drug combinations currently recommend by the WHO can be found in **S1 Table.**

NTD, neglected tropical disease; PK: pharmacokinetic; RCT: randomized controlled trial.

### Choking risk in young children

In the United States, airway obstruction—or choking—is the fourth leading cause of unintentional injury-related deaths in young children, responsible for 0.54 deaths per 100,000 children less than 5 years old in 2019 [48]. Young children are at highest risk due, in large part, to the small diameter of their trachea, ranging from 5 mm in children 1 to 2 years old to 11 mm in those 14 to 18 years old [32]. In general, choking on medication is a relatively infrequent cause of fatal choking, at least in the developed countries where age-appropriate drug formulations are commonly available.

Less is known about incidence of fatal choking among young children in low-resource settings or about the relative importance of medicines as a cause of choking. Age-appropriate drug formulations may be more expensive and less commonly available in these countries [49–51]. Crushing tablets and mixing them with water is a common practice, but clean water may not be available. In a study from Tanzania, where crushing pills for children is common practice, 80% of parents reported problems with administration of medications to their children. For the youngest children, parents preferred oral suspension to crushed tablets [52].

Following a report of 4 choking-related deaths in children during PC with whole albendazole tablets, in 2007 the WHO recommended that manufacturers of anthelminthic drugs for preschool-age children develop “safe single-dose formulations (e.g., granules or liquids for oral use) to replace the tablets currently in use” [18]. Of the 6 drugs currently manufactured under stringent regulatory authority and donated by pharmaceutical companies for NTD PC, azithromycin is available in an oral suspension; a rapidly disintegrating, chewable formulation of mebendazole was recently approved for STH, and an orally dispersible pediatric formulation of praziquantel is in the final stages of development [53–54].

Available evidence, although limited, suggests that forcing a young child to swallow whole tablets is a major risk factor for fatal choking in young children during PC [13,15]. Risk appears to be inversely related to age, since ability to swallow increases with age. Observational assessments during PC suggest that risk of nonfatal choking increases significantly below 3 years of age [13,15]. It is therefore not surprising that, based on available data that are incomplete and often anecdotal, risk of choking during PC for NTDs appears greatest for young children receiving whole deworming tablets, which may be larger than the diameter for the trachea (**Table 1**). The WHO recommends that albendazole tablets be crushed and mixed with water before giving them to young children (defined as children less than 3 years of age), but a recent observational assessment of deworming in children 1 to 2 years of age showed that 24% received whole tablets [13,15].

The availability of oral suspension for trachoma, the new chewable, rapidly disintegrating, mebendazole tablet (donated by Johnson & Johnson), and the forthcoming pediatric formulation of praziquantel open new possibilities for safe coadministration of NTD drugs in young children, specifically with respect to choking [55–61]. However, other questions remain. Appropriate dosing is commonly determined by age (albendazole, DEC, mebendazole), height (ivermectin, praziquantel), or both height and age (azithromycin) [62]. Which drugs can be safely swallowed at the same time? Can a crushed albendazole tablet be added to azithromycin oral suspension? Can the orally dispersible tablets of praziquantel and rapidly disintegrating, chewable tables of mebendazole be mixed with the same water and given at the same time? Even if drugs are given sequentially during the same PC visit, does the order in which they are administered influence acceptability? What safeguards are required to ensure proper dosing? When SAEs do occur, which drug or drugs will be causally implicated in settings of coadministration?

### Survey of current practices and attitudes toward coadministration

Of 15 persons surveyed to assess current practices, 8 (53.3%) work for national Ministries of Health or provincial health bureaus in Africa (5 countries), South America (1 country), or the Western Pacific region (2 countries). The 7 (46.7%) NGO respondents work for 6 different organizations in 10 countries (7 in Africa and 3 in the Americas). Five NGO representatives live in sub-Saharan Africa and the other 2 are based in the United States (**Table 1**). Eight (53.3%) of the 15 respondents reported coadministering drugs not currently recommended for coadministration by the WHO (**Table 4**).

Seven (46.7%) respondents reported that they crushed tablets of albendazole (5 respondents, 33.3%), praziquantel (3 respondents, 20.0%), azithromycin (3 respondents, 20.0%), or mebendazole (1 respondent, 6.7%). Reasons for crushing tablets included the lack of an age-appropriate pediatric formulation of azithromycin for yaws, difficulty swallowing (both for children and adults), the large tablet size of praziquantel, and young children not being able to chew albendazole. Reasons given for not crushing tablets included no perceived difficulty swallowing tablets, lack of clean water, belief that crushing is unnecessary for chewable tablets, the small size of ivermectin tablets, and availability of oral suspension (in the case of azithromycin for trachoma). When giving crushed tablets, 8 (53.3%) respondents reported routinely giving them with water.

Parents are allowed to administer medication during MDA in 11 (73.3%) respondents’ programs. When asked what steps were taken when a child refused to take medicine, 9 (60.0%) reported not forcing the child to do so, 7 (46.7%) reported encouraging the child, 4 (26.7%) reported bringing the child back later, 2 (13.3%) reported offering powder for oral suspension (POS) for azithromycin, and 1 (6.7%) reported having parents or community members intervene to calm the child. In addition, 11 (73.3%) expressed the desirability of coadministering NTD drugs during a single day. Responses did not differ significantly between the 8 respondents working for Ministries of Health and those working for NGOs (**S2 Table**) or between those surveyed by email versus video conferencing (**S3 Table**).

**Table 4:** Common thematic elements of MDA integration surveys.

Topic	Question	Responses	No. (%)
Coadministration	Do you currently practice coadministration for MDAs, with multiple medications given on the same day?	Yes	15 (100.0)
		No	0 (0.0)
	Are these drugs given together at the same time?	Yes	15 (100.0)
		No	0 (0.0)
	What diseases do you treat through coadministration during MDA?	LF + ONC	3 (20.0)
		LF + ONC + STH	2 (13.3)
		LF + ONC + SCH + STH	2 (13.3)
		LF + SCH	2 (13.3)
		LF + SCH + STH	2 (13.3)
		LF + Yaws	1 (6.7)
		Scabies + STH + TRA + Yaws	1 (6.7)
		SCH + STH	3 (20.0)
		STH + TRA	1 (6.7)
	What drug combinations do you coadminister during MDA that are not currently recommended by WHO?	ALB + AZI	2 (13.3)
		ALB + AZI + DEC + IVM	1 (6.7)
		ALB + IVM + PZQ	5 (33.3)
		IVM + MEB	2 (13.3)
		IVM + PZQ	1 (6.7)
Tablet crushing	When treating young children for LF and soil-transmitted helminths during MDA, do you recommend crushing tablets?	Yes	7 (46.7)
		No	8 (53.3)
	If tablets are crushed, is water routinely given in conjunction?	Yes	8 (53.3)
		No	5 (33.3)
	What drugs do you recommend crushing during MDA?	Albendazole	5 (33.3)
		Azithromycin	3 (20.0)
		Mebendazole	1 (6.7)
		Praziquantel	3 (20.0)
Parent involvement	Are parents allowed to give medicine to their children during MDA?	Yes	11 (73.3)
		No	3 (20.0)
	If parents are allowed to give children drugs during MDA, how is treatment observed?	Observed by distribution team	12 (80.0)
		Not observed–parents allowed to take tablets home	2 (13.3)
Child refusal	If a child refuses to take tablets during MDA, what steps are taken?	Encouraging child	7 (46.7)
		Not forcing drug and marking as refusal	9 (60.0)
		Bringing child back	4 (26.7)
		POS offered (for AZI)	2 (13.3)
		Parent/community member calming child	1 (6.7)
NTD integration	If it were safe to give all appropriate NTD drugs during a single day of MDA, would you find that useful?	Yes	11 (73.3)
		No	3 (20.0)
	What drugs would you like to be able to give together during MDA that you currently do not because their coadministration is not recommended by WHO?	ALB + AZI + DEC + IVM	1 (6.7)
		ALB + AZI + IVM	3 (20.0)
		ALB + AZI + IVM + PZQ	1 (6.7)
		ALB + IVM + PZQ	1 (6.7)
		IVM + PZQ	2 (13.3)

ALB, albendazole; AZI, azithromycin; DEC, diethylcarbamazine; IVM, ivermectin; LF, lymphatic filariasis; MEB, mebendazole; MDA, mass drug administration; ONC, onchocerciasis; POS, powder for oral suspension; PZQ, praziquantel; SCH, schistosomiasis; STH, soil-transmitted helminths; TRA, trachoma.

## Discussion

Coadministration of drugs during PC appears to be increasingly common as NTD programs seek greater integration within national health systems. This practice builds on long-standing recommendations for coadministration of 2 (and in some cases, 3) drugs for LF and 2-drug combinations for school-based control of STH and schistosomiasis, as well as the push for integrated PC in the 2012 WHO NTD Roadmap [5]. The survey results suggest that coadministration is a common practice in NTD programs, including with drug combinations that the WHO does not currently recommend. All 5 not-recommended drug combinations that survey respondents reported giving (**Table 4**) have been subjected to clinical safety research (**Table 3**). The number of study subjects receiving these combinations ranged from 2,032 for ivermectin with mebendazole or praziquantel to more than 700,000 for ivermectin, albendazole, and praziquantel. Therefore, safety data exist to support coadministration of drug combinations not yet recommended by WHO.

There are several remaining administrative barriers and safety challenges to expanded use of drug coadministration during MDA. First, safety data on some coadministered NTD drug combinations are not yet sufficient to support official WHO recommendations. Before the WHO approved 2- and 3-drug combinations for LF, pharmacokinetic studies were required, as was enhanced surveillance for adverse reactions in at least 10,000 persons who received these combinations [63,64]. For drug combinations currently being used to address NTDs beyond the 5 diseases typically addressed through PC, such as yaws and scabies, WHO recommendations may require additional pharmacokinetic studies or safety data based on active surveillance.

Second, coadministration potentially increases risk of incorrect dosing and raises practical questions about whether medications should be mixed or crushed together, swallowed at the same time, or given in a particular order. In general, WHO guidelines have not addressed these practical issues, which are handled differently in various settings, and influenced by cultural and other factors. There are few data available on the relative safety or efficiency of different approaches to these questions. Responses to the survey, in which fewer than half of respondents reported routinely crushing tablets for young children, as well an observational assessment by Kernell and colleagues indicate that even in the absence of coadministration, the WHO recommendation to crush tablets for young children is not always followed [15].

Third, coadministration of medicines during the same MDA can mean swallowing them all at the same time or giving them sequentially. Swallowing multiple tablets at the same time could increase risk of refusal or choking, particularly in young children [32]. The degree to which swallowing multiple tablets simultaneously would increase risk of aspiration and obstruction of the trachea is unknown, but would likely be influenced by the participants’ age, formulation of the medicine, the size of the tablets (which is determined by diseases being targeted), whether some or all the medications are crushed, and whether the participant takes the drugs under duress. One way to avoid this potential compounded risk would be to administer the drugs sequentially during the same MDA event.

Fourth, not all drugs given during PC are optimally formulated for young children. The availability of azithromycin as an oral suspension for trachoma elimination and the new rapidly disintegrating, chewable formulation of mebendazole and orally dispersible formulation of praziquantel represent significant advances for MDA safety. Recent data from Ethiopia suggest that risk of choking and other adverse swallowing events is significantly lower among young children who are given azithromycin oral suspension than for children receiving albendazole tablets (whether crushed or not) for STH [15,65]. Therefore, coadministering whole deworming tablets with azithromycin oral suspension may represent a significant increase in choking risk compared to oral suspension alone. However, even for azithromycin, challenges remain. For example, azithromycin is not commonly available as an oral suspension for yaws, and clear guidelines for crushing azithromycin tablets for young children are not available (although, anecdotally, this is being practiced in yaws eradication efforts to reduce choking risk).

Despite these challenges, several recommendations can be made for coadministration of NTD drugs during PC, which are similar to those for administration of individual drugs [16]. Children should not be forced to take medicine, and all treatment should be directly observed by community drug distributors (CDDs) or health personnel [13,16]. Care should be taken during PC to minimize distractions and disorder, since they increase the risk of medical error, such as incorrect dosing [15,66]. CDDs should receive training in appropriate dosing of multiple medications and crowd control and be supported when deciding not to treat children who resist taking medication.

In addition, as highlighted by recent WHO guidance, improved coordination at the central level, for example, with pharmacovigilance agencies and communications specialists, can help to facilitate a culture of safety and to integrate both NTDs and NTD safety into national health systems [16]. Specific areas of focus include enhanced SAE surveillance, response, and reporting, as well as developing action plans and capacity to address unfounded rumors of SAEs quickly and effectively.

Further research is also warranted. Additional monitoring and enhanced surveillance are needed to confirm the safety, tolerability, and acceptability of high-priority drug combinations that are not yet recommended by the WHO. Understanding the social dynamics of swallowing tablets and community norms related to child feeding may yield additional insights into choking prevention.

Where research is missing and unlikely to be prioritized, sharing of preferred practices across programs should be encouraged to address the practical aspects of coadministration and incorporate preferred practices into CDD training [67]. Observational assessments to evaluate current safety practices can be incorporated into supervisory visits and other evaluation exercises.

This inquiry has several limitations. Recommendations to prevent choking during PC are supported by relatively few studies. Our survey of program managers and NGO representatives involved only 15 persons and was based on a convenience sample. Therefore, the results do not provide generalizable global estimates. The use of 2 options for completing the survey—email or teleconference—might have affected the results, although no significant differences were noted.

## Conclusion

Integration of PC for multiple diseases contributes to creating cost-effective, sustainable programs for the control and elimination of NTDs. Coadministration of NTD drugs during PC appears to be increasingly common. Safety of delivering coadministered PC drugs requires attention to choking prevention, use of approved drug combinations, and increased access to age-appropriate drug formulations. Safety can be further reinforced through appropriately training CDDs, not forcing children to take medication, observing all treatments, and minimizing distractions during MDA.

### Key learning points

- The increasingly common practice of coadministering NTD drugs during PC offers significant cost-savings and programmatic benefits and is an example of increased integration of NTD programs within national health systems.
- Coadministration of NTD drugs also presents safety concerns, including potential pharmacological interactions; increased risk of choking while swallowing multiple tablets at the same time; and the possibility of confusion or incorrect dosing when giving drugs with different formulations, exclusion criteria, and recommendations for safe administration.
- Not all drugs given during PC are optimally formulated for young children; fatal choking during MDA has not yet been completely prevented.
- The WHO currently recommends coadministration for a limited number of NTD drugs; further safety monitoring and surveillance are needed to develop recommendations for additional combinations.
- In practice, NTD drug combinations that are not yet recommended by the WHO are being given for PC.

### Top five papers

- World Health Organization. Action Against Worms. PPC Newsletter. 2007(8).
- Kernell JW, DePaola RV, Maglione AM, Ahern LN, Penney NG, Addiss DG. Risk of adverse swallowing events and choking during deworming for preschool-aged children. PLOS Neglected Tropical Diseases. 2018 Jun 22.
- World Health Organization. Promoting safety of medicines for children. Geneva: World Health Organization; 2007.
- World Health Organization. Assuring safety of preventive chemotherapy interventions for the control of neglected tropical diseases. Geneva: World Health Organization; 2011.
- Ciciriello AM, Addiss DG, Teferi T, Emerson PM, Hooper PJ, Seid M, et al. An observational assessment of the safety of mass drug administration for trachoma in Ethiopian children. Transactions of the Royal Society of Tropical Medicine and Hygiene 2022. Advance access publication. doi: https://doi.org/10.1093/trstmh/trac006.

## Supporting information

S1 Appendix Literature search strategy. (DOCX)

S1 Questionnaire Interview guide for partner organizations. (DOCX)

S1 Table Supporting studies for World Health Organization-recommended mass drug administration combinations. **PK**, pharmacokinetic; **RCT**, randomized controlled trial.
(DOCX)

S2 Table Survey on drug coadministration attitudes and practices, by respondent affiliation (Governmental Ministry of Health vs. Non-Governmental Organization). **ALB,** albendazole; **AZI,** azithromycin; **DEC,** diethylcarbamazine; **IVM,** ivermectin; **LF,** lymphatic filariasis; **MDA,** mass drug administration; **MEB,** mebendazole; **MOH**, Ministry of Health; **NGO**, Non-governmental organization; **ONC**, onchocerciasis; **POS**, powder for oral suspension; **PZQ**, praziquantel; **SCH**, schistosomiasis; **STH**, soil-transmitted helminths; **TRA**, trachoma.
(DOCX)

S3 Table Survey on drug coadministration attitudes and practices, by survey platform (e-mail vs. teleconference). **ALB,** albendazole; **AZI,** azithromycin; **DEC,** diethylcarbamazine; **IVM,** ivermectin; **LF,** lymphatic filariasis; **MDA,** mass drug administration; **MEB,** mebendazole; **ONC**, onchocerciasis; **POS,** powder for oral suspension; **PZQ,** praziquantel; **SCH**, schistosomiasis; **STH,** soil-transmitted helminths; **TRA,** trachoma.
(DOCX)

## Abbreviations

CDD: community drug distributor
COVID-19: Coronavirus Disease 2019
DEC: diethylcarbamazine
LF: lymphatic filariasis
MDA: mass drug administration
NGO: non-governmental organization
NTD: neglected tropical disease
PC: preventive chemotherapy
POS: powder for oral suspension
SAE: serious adverse event
STH: soil-transmitted helminthiasis
WHO: World Health Organization

## References

[1] World Health Organization. Summary of global update on implementation of preventive chemotherapy against NTDs in 2020. Wkly Epidemiol Rec. 2021;38:468–75.

[2] Moloo A. Neglected tropical diseases: 2020 preventive chemotherapy treatment coverage declines due to COVID-19 disruptions. 2021 [cited 2021 Oct 14]. In: World Health Organization [Internet]. Available from: https://www.who.int/news/item/24-09-2021-neglected-tropical-diseases-2020-preventive-chemotherapy-treatment-coverage-declines-due-to-covid-19-disruptions.

[3] Richards FO Jr. Upon entering an age of global ivermectin-based mass drug administration for neglected tropical diseases and malaria. Malar J. 2017;16:168. 28438168 10.1186/s12936-017-1830-z PMC5404338

[4] World Health Organization. Ending the neglect to attain the Sustainable Development Goals: a road map for neglected tropical diseases 2021–2030. Geneva: World Health Organization; 2020.

[5] World Health Organization. Accelerating work to overcome the global impact of neglected tropical diseases: a roadmap for implementation: executive summary. Geneva: World Health Organization; 2012.

[6] Smits HL. Prospects for the control of neglected tropical diseases by mass drug administration. Expert Rev Anti Infect Ther. 2009;7(1):37–56. doi: 10.1586/14787210.7.1.37 19622056

[7] Webster JP, Molyneux DH, Hotez PJ, Fenwick A. The contribution of mass drug administration to global health: past, present, and future. Philos Trans R Soc B. 2014;369(1645). 10.1098/rstb.2013.0434 PMC4024227 24821920

[8] Hotez PJ. Mass drug administration and integrated control for the world’s high prevalence neglected tropical diseases. Clin Pharmacol Ther. 2009;85(6):659–664. doi: 10.1038/clpt.2009.16 19322166

[9] Richards FO Jr, Eigege A, Miri ES, Jinadu MY, Hopkins DR. Integration of mass drug administration programs in Nigeria: The challenge of schistosomiasis. Bull World Health Organ. 2006;84(8):673–676. 16917658 10.2471/blt.06.029652 PMC2627425

[10] Bronzan RN, Dorkenoo AM, Agbo YM, Halatoko W, Layibo Y, Adjeloh P, et al. Impact of community-based integrated mass drug administration on schistosomiasis and soil-transmitted helminth prevalence in Togo. PLoS Negl Trop Dis. 2018. doi: 10.1371/journal.pntd.0006551 30125274 PMC6124778

[11] Goldman AS, Brady MA, Direny A, Desir L, Oscard R, Vely J, et al. Costs of integrated mass drug administration for neglected tropical diseases in Haiti. Am J Trop Med Hyg. 2011;85(5):826–833. doi: 10.4269/ajtmh.2011.10-0635 22049035 PMC3205627

[12] Lo NC, Bogoch II, Blackburn BG, Raso G, N’Goran EK, Coulibaly JT, et al. Comparison of community-wide, integrated mass drug administration strategies for schistosomiasis and soil-transmitted helminthiasis: a cost-effectiveness modeling study. Lancet Glob Health. 2015;3:629–638. 10.1016/S2214-109X(15)00047-9 26385302

[13] World Health Organization. Action Against Worms. PPC Newsletter. 2007;8.

[14] World Health Organization. Assuring safety of preventive chemotherapy interventions for the control of neglected tropical diseases. Geneva: World Health Organization; 2011.

[15] Kernell JW, DePaola RV, Maglione AM, Ahern LN, Penney NG, Addiss DG. Risk of adverse swallowing events and choking during deworming for preschool-aged children. PLoS Negl Trop Dis. 2018. doi: 10.1371/journal.pntd.0006578 29933362 PMC6014639

[16] World Health Organization. Safety in administering medicines for neglected tropic diseases. Geneva: World Health Organization; 2021.

[17] World Health Organization. How to add deworming to vitamin A distribution. Geneva: World Health Organization; 2004.

[18] World Health Organization. Promoting safety of medicines for children. Geneva: World Health Organization; 2007.

[19] World Health Organization. Lymphatic filariasis–Status of Mass Drug Administration: 2020. 2020 [cited 2021 Apr 19]. In: World Health Organization [Internet]. Available from: https://apps.who.int/neglected_diseases/ntddata/lf/lf.html.

[20] World Health Organization. Onchocerciasis–Status of endemicity of onchocerciasis: 2019. 2020 [cited 2021 Apr 19]. In: World Health Organization [Internet]. Available from: https://apps.who.int/neglected_diseases/ntddata/oncho/onchocerciasis.html.

[21] World Health Organization. Schistosomiasis–Status of endemic countries: 2020. 2020 [cited 2021 Apr 19]. In: World Health Organization [Internet]. Available from: https://apps.who.int/neglected_diseases/ntddata/sch/sch.html.

[22] World Health Organization. Soil-transmitted helminthiases–Number of children (Pre-SAC and SAC) requiring preventative chemotherapy for soil-transmitted helminthiases: 2019. 2020 [cited 2021 Apr 19]. In: World Health Organization [Internet]. Available from: https://apps.who.int/neglected_diseases/ntddata/sth/sth.html.

[23] World Health Organization. Trachoma–Status of elimination of trachoma as a public health problem: 2020. 2020 [cited 2021 Apr 19]. In: World Health Organization [Internet]. Available from: https://apps.who.int/neglected_diseases/ntddata/trachoma/trachoma.html.

[24] World Health Organization. Diseases–Schistosomiasis. 2019 [cited 2021 Apr 19]. In: World Health Organization [Internet]. Available from: https://espen.afro.who.int/diseases/schistosomiasis.

[25] World Health Organization. Diseases–Soil-transmitted helminthiasis. 2019 [cited 2021 Apr 19]. In: World Health Organization [Internet]. Available from: https://espen.afro.who.int/diseases/soil-transmitted-helminthiasis.

[26] World Health Organization. Helminth control in school-age children: a guide for managers of control programmes. 2nd ed. Geneva: World Health Organization; 2011.

[27] World Health Organization. Control of Neglected Tropical Diseases. 2022. [cited 2022 Sep 8]. In: World Health Organization [Internet]. Available from: https://www.who.int/teams/control-of-neglected-tropical-diseases/lymphatic-filariasis/global-programme-to-eliminate-lymphatic-filariasis.

[28] World Health Organization. Lymphatic filariasis. 2022 [cited 2022 Sep 8]. In: World Health Organization [Internet]. Available from: https://www.who.int/news-room/fact-sheets/detail/lymphatic-filariasis.

[29] World Health Organization. Guideline: alternative mass drug administration regimens to eliminate lymphatic filariasis. Geneva: World Health Organization; 2017. 29565523

[30] World Health Organization. Schistosomiasis and soil-transmitted helminthiases: numbers of people treated in 2019. Wkly Epidemiol Rec. 2020;95(50):629–40.

[31] World Health Organization. Global programme to eliminate lymphatic filariasis: progress report, 2019. Wkly Epidemiol Rec. 2020;95(43):509–24.

[32] Amendola RL, Reinhardt JM, Zimmerman MB, Sato Y, Diggelmann HR, Kacmarynski DS. Development of a preliminary pediatric tracheal growth model from magnetic resonance images. Laryngoscope. 2014;124(8):1947–1951. doi: 10.1002/lary.24547 24307560

[33] World Health Organization. WHO Alliance for the Global Elimination of Trachoma by 2020: progress report on elimination of trachoma, 2020. Wkly Epidemiol Rec. 2021;96(31):353–64.

[34] PopulationPyramid.net. Population Pyramids of the World from 1950 to 2100: Ethiopia 2020. 2019 [cited 2022 Feb 2]. In: Creative Commons [Internet]. Available from: https://www.populationpyramid.net/ethiopia/2020/.

[35] Eigege A, Pede E, Miri E, Umaru E, Ogbu Pearce P, Jinadu MY, et al. Triple drug administration (TDA), with praziquantel, ivermectin and albendazole, for the prevention of three neglected tropical diseases in Nigeria. Ann Trop Med Parasitol. 2008;102(2):177–179. doi: 10.1179/136485908X252322 18318940

[36] Mohammed KA, Haji HJ, Gabrielli A, Mubila L, Biswas G, Chitsulo L, et al. Triple Co-Administration of Ivermectin, Albendazole and Praziquantel in Zanzibar: A Safety Study. PLOS Negl Trop Dis. 2008;2(1). doi: 10.1371/journal.pntd.0000171 18235853 PMC2217668

[37] Na-Bangchang K, Kietinun S, Pawa KK, Hanpitakpong W, Na-Bangchang C, Lazdins J. Assessments of pharmacokinetic drug interactions and tolerability of albendazole, praziquantel and ivermectin combinations. Trans R Soc Trop Med Hyg. 2006;100:335–345. doi: 10.1016/j.trstmh.2005.05.017 16271272

[38] Namwanje H, Kabatereine N, Olsen A. A randomised controlled clinical trial on the safety of co-administration of albendazole, ivermectin and praziquantel in infected schoolchildren in Uganda. Trans R Soc Trop Med Hyg. 2011;105:181–88. doi: 10.1016/j.trstmh.2010.11.012 21353271

[39] Marks M, Toloka H, Baker C, Kositz C, Asugeni J, Puiahi E, et al. Randomized Trial of Community Treatment With Azithromycin and Ivermectin Mass Drug Administration for Control of Scabies and Impetigo. Clin Infect Dis. 2019;68(6):927–33. doi: 10.1093/cid/ciy574 29985978 PMC6399435

[40] Romani L, Marks M, Sokana O, Nasi T, Kamoriki B, Wand H, et al. Feasibility and safety of mass drug coadministration with azithromycin and ivermectin for the control of neglected tropical diseases: a single-arm interventional trial. Lancet Glob Health. 2018;6. 10.1016/S2214-109X(18)30397-8 PMC6139784 30223985

[41] John LN, Bjerum C, Millat Martinez P, Likia R, Silus L, Wali C, et al. Pharmacokinetic and safety study of co-administration of albendazole, diethylcarbamazine Ivermectin and azithromycin for the integrated treatment of Neglected Tropical Diseases. Clin Infect Dis. 2020. doi: 10.1093/cid/ciaa1202 32818264

[42] John LN, Gonzalez-Beiras C, Vall-Mayans M, Kolmau R, Houinei W, Wangi J, et al. Safety of mass drug coadministration with ivermectin, diethylcarbamazine, albendazole, and azithromycin for the integrated treatment of neglected tropical diseases: a cluster randomized community trial. Lancet Reg Health West Pac. 2021;19(29). 10.1016/j.lanwpc.2021.100293 PMC8661048 35024646

[43] Amsden GW, Gregory TB, Michalak CA, Glue P, Knirsch CA. Pharmacokinetics of azithromycin and the combination of ivermectin and albendazole when administered alone and concurrently in health volunteers. Am J Trop Med Hyg. 2007;76(76):1153–1157. 17556628

[44] Coulibaly YI, Dicko I, Keita M, Keita MM, Doumbia M, Daou A, et al. A Cluster Randomized Study of The Safety of Integrated Treatment of Trachoma and Lymphatic Filariasis in Children and Adults in Sikasso, Mali. PLoS Negl Trop Dis. 2013;7(5). doi: 10.1371/journal.pntd.0002221 23675549 PMC3649960

[45] El-Tahwaty A, Glue P, Andrews EN, Mardekian J, Amsden GW, Knirsch CA. The Effect of Azithromycin on Ivermectin Pharmacokinetics–A Population Pharmacokinetic Model Analysis. PLoS Negl Trop Dis. 2008;2. 10.1371/journal.pntd.0000236 PMC2359853 18478051

[46] Ndyomugyenyi R, Kabatereine N. Integrated community-directed treatment for the control of onchocerciasis, schistosomiasis and intestinal helminths infections in Uganda: advantages and disadvantages. Trop Med Int Health. 2003;8(11):997–1004. doi: 10.1046/j.1360-2276.2003.01124.x 14629766

[47] Speich B, Ali SM, Ame SM, Bogoch II, Alles R, Huwyler J, et al. Efficacy and safety of albendazole plus ivermectin, albendazole plus mebendazole, albendazole plus oxantel pamoate, and mebendazole alone against Trichuris trichiura and concomitant soil-transmitted helminth infections: a four-arm, randomised controlled trial. Lancet Infect Dis. 2015;15:277–284. doi: 10.1016/S1473-3099(14)71050-3 25589326

[48] National Safety Council. Deaths by Age and Cause. 2021 [cited 2021 Dec 1]. In: National Safety Council [Internet]. Available from: https://injuryfacts.nsc.org/all-injuries/deaths-by-demographics/deaths-by-age/data-details/.

[49] Craig SR, Adams LV, Spielberg SP, Campbell B. Pediatric therapeutics and medicine administration in resource-poor settings: a review of barriers and an agenda for interdisciplinary approaches to improving outcomes. Soc Sci Med. 2009;69(11):1681–1690. doi: 10.1016/j.socscimed.2009.08.024 19796859

[50] Hoppu K, Sri Ranganathan S, Dodoo AN. Realities of paediatric pharmacotherapy in the developing world. Arch Dis Child. 2011;96(8):764–768. doi: 10.1136/adc.2009.180000 21441240

[51] Sosnik A, Seremeta KP, Imperiale JC, Chiappetta DA. Novel formulation and drug delivery strategies for the treatment of pediatric poverty-related diseases. Expert Opin Drug Deliv. 2012;9(3):303–323. doi: 10.1517/17425247.2012.655268 22257003

[52] Adams LV, Craig SR, John Mmbaga E, Naburi H, Lahey T, Nutt CT, et al. Children’s medicines in Tanzania: a national survey of administration practices and preferences. PLoS ONE. 2018;8(3). 10.1371/journal.pone.0058303 PMC3590153 23484012

[53] Global Health Innovative Technology Fund. GHIT and EDCTP co-invest additional 7.8 million Euro in the Pediatric Praziquantel Consortium’s access program to ensure treatment of schistosomiasis for preschool-aged children. 2021 [cited 2021 Apr 19]. In: Global Health Innovative Technology Fund [Internet]. Available from: https://www.ghitfund.org/newsroom/press/detail/302/.

[54] Pediatric Praziquantel Consortium. The pediatric formulation. 2021 [cited 2021 Apr 19]. In: Pediatric Praziquantel Consortium [Internet]. Available from: https://www.pediatricpraziquantelconsortium.org/node/36.

[55] Bagchus WM, Bezuidenhout D, Harrison-Moench E, Kourany-Lefoll E, Wolna P, Yalkinoglu O. Bioavailability of Orally Dispersible Tablet Formulations of Levo- and Racemic Praziquantel: Two Phase I Studies. Clin Transl Sci. 2019;12(1):66–76. 30536632 10.1111/cts.12601 PMC6342245

[56] Friedman AJ, Ali SM, Albonico M. Safety of a New Chewable Formulation of Mebendazole for Preventative Chemotherapy Interventions to Treat Young Children in Countries with Moderate-to-High Prevalence of Soil Transmitted Helminth Infections. J Trop Med. 2012;2012. 10.1155/2012/590463 PMC3540782 23319961

[57] Navaratnam AM, Sousa-Figueiredo JC, Stothard JR, Kabatereine NB, Fenwick A, Mutumba-Nakalembe MJ. Efficacy of praziquantel syrup versus crushed praziquantel tablets in the treatment of intestinal schistosomiasis in Ugandan preschool children, with observation on compliance and safety. Trans R Soc Trop Med Hyg. 2012;106(7):400–407. doi: 10.1016/j.trstmh.2012.03.013 22657533

[58] Palmeirim MS, Bosch F, Ame SM, Ali SM, Hattendorf J, Keiser J. Efficacy, safety and acceptability of a new chewable formulation versus the solid tablet of mebendazole against hookworm infection in children: An open-label, randomized controlled trial. EClinicalMedicine. 2020; 27. 10.1016/j.eclinm.2020.100556 PMC7599302 33150325

[59] Silber SA, Diro E, Workneh N, Mekonnen Z, Levecke B, Steinmann P, et al. Efficacy and Safety of a Single-Dose Mebendazole 500 mg Chewable, Rapidly-Disintegrating Tablet for Ascaris lumbricoides and Trichuris trichiura Infection Treatment in Pediatric Patients: A Double-Blind, Randomized, Placebo-Controlled, Phase 3 Study. Am J Trop Med Hyg. 2017;97(6):1851–56. doi: 10.4269/ajtmh.17-0108 29016336 PMC5805036

[60] Trastullo R, Dolci LS, Passerini N, Albertini B. Development of flexible and dispersible oral formulations containing praziquantel for potential schistosomiasis treatment of pre-school age children. Int J Pharm. 2015;495(1):536–550. doi: 10.1016/j.ijpharm.2015.09.019 26386139

[61] Webb EL, Edielu A, Wu HW, Kabatereine NB, Tukahebwa EM, Mubangizi A, et al. The praziquantel in preschoolers (PIP) trial: study protocol for a phase II PK/PD-driven randomised controlled trial of praziquantel in children under 4 years of age. Trials. 2021;22(1). doi: 10.1186/s13063-021-05558-1 34488846 PMC8419815

[62] International Trachoma Initiative. Zithromax Management Guide 2019. International Trachoma Initiative; 2019.

[63] McLaughlin SI, Radday J, Michel MC, Addiss DG, Beach MJ, Lammie PJ, et al. Frequency, severity, and costs of adverse reactions following mass treatment for lymphatic filariasis using diethylcarbamazine and albendazole in Leogane, Haiti, 2000. Am J Trop Med Hyg. 2003;68(5):568–573. doi: 10.4269/ajtmh.2003.68.568 12812348

[64] Weil GJ, Bogus J, Christian M, Dubray C, Djuardi Y, Fischer PU, et al. The safety of double- and triple-drug community mass drug administration for lymphatic filariasis: A multicenter, open-label, cluster-randomized study. PLoS Med. 2019;16(6). doi: 10.1371/journal.pmed.1002839 31233507 PMC6590784

[65] Ciciriello AM, Addiss DG, Teferi T, Emerson PM, Hooper PJ, Seid M, et al. An observational assessment of the safety of mass drug administration for trachoma in Ethiopian children. Trans R Soc Trop Med Hyg. Advance access publication. 2022. doi: 10.1093/trstmh/trac006 35106593 PMC9526842

[66] Kohn LT, Corrigan JM, Donaldson MS, editors. To err is human: Building a safer health system. Washington, DC: National Academy Press; 1999. 25077248

[67] Addiss D, Sarah V, Negussu N, Emerson P. Safe Mass Drug Administration and Trachoma Elimination. Community Eye Health. 2019;32(106):37. 31649434 PMC6802476

