# Supplementary material for: Safety of integrated preventive chemotherapy for neglected tropical diseases
Source: PLoS Negl Trop Dis. 2022 Sep 29;16(9):e0010700. doi: 10.1371/journal.pntd.0010700 (PMC9521808; doi:10.1371/journal.pntd.0010700)
Supplement: S1 Appendix — (DOCX) [file pntd.0010700.s001.docx]

**Safety of co-administered neglected tropical disease drugs**

The following search strings were used on Google Scholar, PubMed, Cochrane, Scopus and ClinicalTrials.gov to identify relevant neglected tropical disease drug studies:

1. albendazole AND (azithromycin OR diethylcarbamazine OR DEC OR ivermectin OR mebendazole OR praziquantel)
2. albendazole AND (diethylcarbamazine OR DEC OR ivermectin OR praziquantel) AND safety
3. albendazole AND (diethylcarbamazine OR DEC) AND ivermectin AND safety
4. azithromycin AND (diethylcarbamazine OR DEC OR ivermectin OR mebendazole OR praziquantel)
5. (diethylcarbamazine OR DEC) AND (ivermectin OR mebendazole OR praziquantel)
6. ivermectin AND (mebendazole OR praziquantel)
7. mebendazole AND praziquantel
8. mebendazole AND praziquantel AND safety

Official dosing and control program guidance documents were also collected from the World Health Organization website for lymphatic filariasis, onchocerciasis, schistosomiasis, soil-transmitted helminthiasis, and trachoma.

**Choking risks in young children**

Choking on medications in preschool children

The following specific PubMed search strings were used to research the epidemiology of choking on medications in preschool children:

1. “airway obstruction/epidemiology" AND "airway obstruction/etiology" AND child
2. ((choke* OR choking OR airway obstruction OR aspiration) AND (medication OR pill* OR tablet* OR capsule*) AND (child OR preschool) AND (epidemiology OR case reports OR incidence)) AND foreign body
3. ("Airway Obstruction/epidemiology” AND "Airway Obstruction/etiology") AND children AND (pill* OR tablet* OR capsule* OR chewable), ((choke* OR choking OR airway obstruction OR aspiration) AND (medication OR pill* OR tablet* OR capsule*) AND (child OR preschool) AND (epidemiology OR case reports OR incidence))
4. ((choke* OR choking OR airway obstruction OR aspiration) AND (medication OR pill* OR tablet* OR capsule*) AND (child OR preschool) AND (epidemiology OR case reports OR incidence))

In Embase, the search string for the first question was as follows: 'airway obstruction'/exp OR choking OR choke* AND ('pill'/exp OR 'drug capsule'/exp OR 'tablet'/exp) AND (incidence OR epidemiology) AND children.

Administering pills to children under 5 years of age

To identify best practices for administering pills to children under 5 years, including magistral dosing, we reviewed published WHO guidelines and conducted searches in PubMed and Embase using the following search strings:

PubMed: (dosage forms OR drug administration routes) AND (extemporaneous OR magistral OR crush OR crushed OR pulverize OR pulverized OR dissolve OR dissolved); limit results to child

Embase: ('drug dosage form'/exp OR 'drug administration route'/exp) AND (extemporaneous OR magistral OR crush* OR pulverize* OR dissolve*), limit to preschool aged children
