# Supplementary material for: Safety of integrated preventive chemotherapy for neglected tropical diseases
Source: PLoS Negl Trop Dis. 2022 Sep 29;16(9):e0010700. doi: 10.1371/journal.pntd.0010700 (PMC9521808; doi:10.1371/journal.pntd.0010700)
Supplement: S1 Questionnaire — (DOCX) [file pntd.0010700.s002.docx]

Interview Guide for Partner Organizations

1. Do you currently practice co-administration for mass drug administrations with multiple medications being given on the same day?
2. Are drugs that are given the same day during MDAs given together at the same time?
3. What diseases do you treat through co-administration during MDAs?
4. What combinations of drugs do you distribute for co-administrations during MDAs?
5. When treating young children for lymphatic filariasis and soil-transmitted helminths during MDAs, do you recommend crushing tablets?
6. If tablets are crushed during MDAs, is water routinely given in conjunction?
7. What drugs do you recommend crushing if this is implemented during an MDA?
8. Are parents allowed to give medicine to their children during MDAs?
9. If parents are allowed to give children drugs during an MDA, how is this done (i.e. – taking the medication homes, only when observed by a health worker, given at the MDA site without health worker observation, etc.)?
10. If a child refuses to take tablets during an MDA, what steps are taken?
11. If it were safe to give all appropriate NTD drugs during a single day of MDA, would you find that useful?
12. What drugs would you like to be able to give together during MDAs that you currently don’t because their co-administration is not recommended by WHO?
