# Supplementary material for: Safety of integrated preventive chemotherapy for neglected tropical diseases
Source: PLoS Negl Trop Dis. 2022 Sep 29;16(9):e0010700. doi: 10.1371/journal.pntd.0010700 (PMC9521808; doi:10.1371/journal.pntd.0010700)
Supplement: S1 Table — PK, pharmacokinetic; RCT, randomized controlled trial. (DOCX) [file pntd.0010700.s003.docx]

| **Author** | **Title** | **Type of Study** | **No. Subjects** | **Location** |
| --- | --- | --- | --- | --- |
| **Albendazole + DEC** | | | | |
| Dubray CL et al. [1] | Safety and efficacy of co-administered diethylcarbamazine, albendazole and ivermectin during mass drug administration  for lymphatic filariasis in Haiti: Results from a two-armed, open-label, cluster-randomized, community study | RCT | 2,994 | Haiti |
| Hardy M et al. [2] | The safety of combined triple drug therapy with ivermectin, diethylcarbamazine and albendazole in the neglected tropical diseases co-endemic setting of Fiji: A cluster randomised trial | RCT | 1,338 | Fiji |
| Jamboulingam et al. [3] | An open label, block randomized, community study of the safety and efficacy of coadministered ivermectin, diethylcarbamazine plus albendazole vs. diethylcarbamazine plus albendazole for lymphatic filariasis in India | RCT | 4,160 | India |
| Khaemba C et al. [4] | Safety and Tolerability of Mass Diethylcarbamazine and  Albendazole Administration for the Elimination of Lymphatic  Filariasis in Kenya: An Active Surveillance Study | Safety | 10,010 | Kenya |
| Kshirsagar NA et al. [5] | Safety, tolerability, efficacy and plasma concentrations of diethylcarbamazine and albendazole co-administration in a field study in an area endemic for lymphatic filariasis in India | RCT | 78 | India |
| Ismail MM et al. [6] | Efficacy of single dose combinations of albendazole, ivermectin and diethylcarbamazine for the treatment of bancroftian filariasis | RCT | 13 | Sri Lanka |
| Ismail MM et al. [7] | Efficacy of albendazole and its combinations with ivermectin or diethylcarbamazine (DEC) in the treatment of *Trichuris trichiura* infections in Sri Lanka | RCT | 47 | Sri Lanka |
| McLaughlin SI et al. [8] | Frequency, severity, and costs of adverse reactions following mass treatment for lymphatic filariasis using diethylcarbamazine and albendazole in Leogane, Haiti, 2000 | Safety | 71,187 | Haiti |
| Shenoy RK et al. [9] | The pharmacokinetics, safety and tolerability of the co-administration of diethylcarbamazine and albendazole | RCT | 14 | India |
| Tavul L et al. [10] | Safety and efficacy of mass drug administration with a single-dose triple-drug regimen of albendazole + diethylcarbamazine + ivermectin for lymphatic filariasis in Papua New Guinea: An open-label, cluster randomised trial | RCT | 2,181 | Papua New Guinea |
| Thomsen EK et al. [11] | Efficacy, Safety, and Pharmacokinetics of Coadministered Diethylcarbamazine, Albendazole, and Ivermectin for Treatment of Bancroftian Filariasis | RCT | 12 | Papua New Guinea |
| Weil GJ et al. [12] | The safety of double- and triple-drug community mass drug administration for lymphatic filariasis: A multicenter, open-label, cluster-randomized study | RCT | 12,280 | Haiti, Fiji, India, Indonesia, Papua New Guinea |
| Yongyuth P et al. [13] | Adverse reactions of 300 MG diethylcarbamazine, and in a combination of 400 MG albendazole, for a mass annual single dose treatment, in migrant workers in Phang Nga province | RCT | 150 | Myanmar |
| **Albendazole + Ivermectin** | | | | |
| Asio SM et al. [14] | Mansonella perstans: safety and efficacy of ivermectin alone, albendazole alone and the two drugs in combination | Safety | 15 | Uganda |
| Awadzi K et al. [15] | The co-administration of ivermectin and albendazole – safety, pharmacokinetics and efficacy against *Onchocerca volvulus* | RCT | 14 | Ghana |
| Bjerum CM et al. [16] | Efficacy and Safety of a Single Dose of Ivermectin, Diethylcarbamazine, and Albendazole for Treatment of Lymphatic Filariasis in Côte d’Ivoire: An Open-label Randomized Controlled Trial | RCT | 52 | Côte d’Ivoire |
| Hürlimann E et al. [17] | Efficacy and safety of co-administered ivermectin and albendazole in school-aged children and adults infected with *Trichuris trichiura* in Côte d’Ivoire, Laos, and Pemba Island, Tanzania: a double-blind, parallel-group, phase 3, randomised controlled trial | RCT | 838 | Côte d’Ivoire, Laos, and Tanzania |
| Ismail MM et al. [6] | Efficacy of single dose combinations of albendazole, ivermectin and diethylcarbamazine for the treatment of bancroftian filariasis | RCT | 13 | Sri Lanka |
| Ismail MM et al. [7] | Efficacy of albendazole and its combinations with ivermectin or diethylcarbamazine (DEC) in the treatment of *Trichuris trichiura* infections in Sri Lanka | RCT | 53 | Sri Lanka |
| Keiser PB et al. [18] | Clinical characteristics of post-treatment reactions to ivermectin/albendazole for *Wuchereria bancrofti* in a region co-endemic for *Mansonella perstans* | Safety | 143 | Mali |
| Makunde WH et al. [19] | Treatment of co-infection with bancroftian filariasis and  onchocerciasis: a safety and efficacy study of albendazole with ivermectin compared to treatment of single infection with bancroftian filariasis | RCT | 27 | Tanzania |
| Matamoros G et al. [20] | Efficacy and Safety of Albendazole and High-Dose Ivermectin Coadministration in School-Aged Children Infected With Trichuris trichiura in Honduras: A Randomized Controlled Trial | RCT | 115 | Honduras |
| Ndyomugyenyi et al. [21] | Efficacy of ivermectin and albendazole alone and in combination for treatment of soil-transmitted helminths in pregnancy and adverse events: a randomized open label controlled intervention trial in Masindi district, western Uganda | RCT | 199 | Uganda |
| **Albendazole + DEC + Ivermectin** | | | | |
| Bjerum CM et al. [16] | Efficacy and Safety of a Single Dose of Ivermectin, Diethylcarbamazine, and Albendazole for Treatment of Lymphatic Filariasis in Côte d’Ivoire: An Open-label Randomized Controlled Trial | RCT | 43 | Côte d’Ivoire |
| Dubray CL et al. [1] | Safety and efficacy of co-administered diethylcarbamazine, albendazole and ivermectin during mass drug administration  for lymphatic filariasis in Haiti: Results from a two-armed, open-label, cluster-randomized, community study | RCT | 3,004 | Haiti |
| Edi C et al. [22] | Pharmacokinetics, safety, and efficacy of a single co-administered dose of diethylcarbamazine, albendazole and  ivermectin in adults with and without Wuchereria bancrofti infection in Côte d’Ivoire | Safety | 56 | Côte d’Ivoire |
| Hardy M et al. [2] | The safety of combined triple drug therapy with ivermectin, diethylcarbamazine and albendazole in the neglected tropical diseases co-endemic setting of Fiji: A cluster randomised trial | RCT | 2,274 | Fiji |
| Jamboulingam et al. [3] | An open label, block randomized, community study of the safety and efficacy of coadministered ivermectin, diethylcarbamazine plus albendazole vs. diethylcarbamazine plus albendazole for lymphatic filariasis in India | RCT | 4,758 | India |
| Tavul L et al. [10] | Safety and efficacy of mass drug administration with a single-dose triple-drug regimen of albendazole + diethylcarbamazine + ivermectin for lymphatic filariasis in Papua New Guinea: An open-label, cluster randomised trial | RCT | 2,382 | Papua New Guinea |
| Thomsen EK et al. [11] | Efficacy, Safety, and Pharmacokinetics of Coadministered Diethylcarbamazine, Albendazole, and Ivermectin for Treatment of Bancroftian Filariasis | RCT | 12 | Papua New Guinea |
| Weil GJ et al. [12] | The safety of double- and triple-drug community mass drug administration for lymphatic filariasis: A multicenter, open-label, cluster-randomized study | RCT | 14,556 | Haiti, Fiji, India, Indonesia, Papua New Guinea |
| **Albendazole + Praziquantel** | | | | |
| Garcia HH et al. [23] | Pharmacokinetics of combined treatment with praziquantel and albendazole in neurocysticercosis | PK | 16 | Peru |
| Garcia HH et al. [24] | Efficacy of combined antiparasitic therapy with praziquantel and albendazole for neurocysticercosis: a double-blind, randomized controlled trial | RCT | 41 | Peru |
| Kaur S et al. [25] | Combination Therapy With Albendazole and Praziquantel Versus Albendazole Alone in Children With Seizures and Single Lesion Neurocysticercosis | RCT | 53 | India |
| Lima RM et al. [26] | Albendazole-praziquantel interaction in healthy volunteers: kinetic disposition, metabolism and enantioselectivity | PK | 9 | Brazil |
| Njenga SM et al. [27] | A School-Based Cross-Sectional Survey of Adverse Events following Co-Administration of Albendazole and Praziquantel for Preventive Chemotherapy against Urogenital Schistosomiasis and Soil-Transmitted Helminthiasis in Kwale County, Kenya | Safety | 752 | Kenya |
| Olds GR et al. [28] | Double-Blind Placebo-Controlled Study of Concurrent Administration of Albendazole and Praziquantel in Schoolchildren with Schistosomiasis and Geohelminths | RCT | 392 | China, Kenya, Philippines |
| Pengsaa K et al. [29] | Pharmacokinetic investigation of albendazole and praziquantel in Thai children infected with *Giardia intestinalis* | PK | 10 | Thailand |
| **Mebendazole + Praziquantel** | | | | |
| Namwanje H et al. [30] | The acceptability and safety of praziquantel alone and in combination with mebendazole in the treatment of Schistosoma mansoni and soil-transmitted helminthiasis in children aged 1–4 years in Uganda | RCT | 41 | Uganda |

1. Dubray CL, Sircar AD, Beau de Rochars, Valery Madsen, Bogus J, Direny AN, Ernest JR, et al. Safety and efficacy of co-administered diethylcarbamazine, albendazole and ivermectin during mass drug administration for lymphatic filariasis in Haiti: Results from a two-armed, open-label, cluster-randomized, community study. PLOS Neglected Tropical Diseases. 2020 Jun 8;14(6):1-21.
2. Hardy M, Samuela J, Kama M, Tuicakau M, Romani L, Whitfeld MJ, et al. The safety of combine triple drug therapy with ivermectin, diethylcarbamazine and albendazole in the neglected tropical diseases co-endemic setting of Fiji: A cluster randomised trial. PLOS Neglected Tropical Diseases. 2020 Mar 16;14(3):1-17.
3. Jambulingam P, Kuttiatt VS, Krishnamoorthy K, Subramanian S, Srividya A, Raju HKK, et al. An open label, block randomized, community study of the safety and efficacy of coadministered ivermectin, diethylcarbamazine plus albendazole vs. diethylcarbamazine plus albendazole for lymphatic filariasis in India. PLOS Neglected Tropical Diseases. 2021 Feb 16;15(2):1-26.
4. Khaemba C, Barry A, Omondi WP, Bota K, Matendechero S, Wandera C, et al. Safety and Tolerability of Mass Diethylcarbamazine and Albendazole Administration for the Elimination of Lymphatic Filariasis in Kenya: An Active Surveillance Study. Pharmaceuticals. 2021 Mar 15;14(264):1-15.
5. Kshirsagar NA, Gogtay NJ, Deshmukh PR, Rajgor DD, Kadam VS, Kirodian BG, et al. Safety, tolerability, efficacy and plasma concentrations of diethylcarbamazine and albendazole co-administration in a field study in an area endemic for lymphatic filariasis in India. Transactions of the Royal Society of Tropical Medicine and Hygiene. 2004 Apr;98(4):205-17.
6. Ismail MM, Jayakody RL, Weil GJ, Nirmalan N, Jayasinghe KSA, Abeyewickrema W, et al. Efficacy of single dose combinations of albendazole, ivermectin and diethylcarbamazine for the treatment of bancroftian filariasis. Transactions of the Royal Society of Tropical Medicine and Hygiene. 1998;92:93-7.
7. Ismail MM, Jayakody RL. Efficacy of albendazole and its combinations with ivermectin or diethylcarbamazine (DEC) in the treatment of *Trichuris trichiura* infections in Sri Lanka. Annals of Tropical Medicine & Parasitology. 1999;93(5):501-4.
8. McLaughlin SI, Radday J, Michel MC, Addiss DG, Beach MJ, Lammie PJ, et al. Frequency, severity, and costs of adverse reactions following mass treatment for lymphatic filariasis using diethylcarbamazine and albendazole in Leogane, Haiti, 2000. American Journal of Tropical Medicine and Hygiene. 2003;68(5):568-73.
9. Shenoy RK, Suma TK, John A, Arun SR, Kumaraswami V, Fleckenstein LL, et al. The pharmacokinetics, safety and tolerability of the co-administration of diethylcarbamazine and albendazole. Annals of Tropical Medicine & Parasitology. 2002;96(6):603-14.
10. Tavul L, Laman M, Howard C, Kotty B, Samuel A, Bjerum C, et al. Safety and efficacy of mass drug administration with a single-dose triple-drug regimen of albendazole + diethylcarbamazine + ivermectin for lymphatic filariasis in Papua New Guinea: An open-label, cluster randomised trial. PLOS Neglected Tropical Diseases. 2022 Feb 9;16(2):1-18.
11. Thomsen EK, Sanuku N, Baea M, Satofan S, Maki E, Lombore B, et al. Efficacy, Safety, and Pharmacokinetics of Coadministered Diethylcarbamazine, Albendazole, and Ivermectin for Treatment of Bancroftian Filariasis. Clinical infectious Diseases. 2016 Feb 1;62(3):334-41.
12. Weil GJ, Bogus J, Christian M, Dubray C, Djuardi Y, Fischer PU, et al. The safety of double- and triple-drug community mass drug administration for lymphatic filariasis: A multicenter, open-label, cluster-randomized study. PLOS Medicine. 2019 Jun 24;16(6):1-20.
13. Yongyuth P, Koyadun S, Jaturabundit N, Jariyahuttakij W, Bhumiratana A. Adverse reactions of 300 MG diethylcarbamazine, and in a combination of 400 MG albendazole, for a mass annual single dose treatment, in migrant workers in Phang Nga province. Journal of the Medical Association of Thailand. 2007 Mar;90(3):552-63.
14. Asio SM, Simonsen PE, Onapa AW. Mansonella perstans: safety and efficacy of ivermectin alone, albendazole alone and the two drugs in combination. Annals of Tropical Medicine and Parasitology. 2009 Jan;103(1):31-7.
15. Awadzi K, Edwards G, Duke BOL, Opoku NO, Attah SK, Addy ET, et al. The co-administration of ivermectin and albendazole – safety, pharmacokinetics and efficacy against *Onchocerca volvulus*. Annals of Tropical Medicine & Parasitology. 2003;97(2):165-78.
16. Bjerum CM, Ouattara AF, Aboulaye M, Kouadio O, Marius VK, Andersen BJ, et al. Efficacy and Safety of a Single Dose of Ivermectin, Diethylcarbamazine, and Albendazole for Treatment of Lymphatic Filariasis in Côte d’Ivoire: An Open-label Randomized Controlled Trial. Clinical Infectious Diseases. 2020 Oct 1;71(7):68-75.
17. Hurlimann E, Keller L, Patel C, Welsche S, Hattendorf J, Ali SM, et al. Efficacy and safety of co-administered ivermectin and albendazole in school-aged children and adults infected with *Trichuris trichiura* in Côte d’Ivoire, Laos, and Pemba Island, Tanzania: a double-blind, parallel-group, phase 3, randomised controlled trial. Lancet Infectious Diseases. 2022 January;22:123-35.
18. Keiser PB, Coulibaly YI, Keita F, Traore D, Diallo A, Diallo DA, et al. Clinical characteristics of post-treatment reactions to ivermectin/albendazole for *Wuchereria bancrofti* in a region co-endemic for *Mansonella perstans*. American Journal of Tropical Medicine and Hygiene. 2003;69(3):331-5.
19. Makunde WH, Kamugisha LM, Massaga JJ, Makunde RW, Savael ZX, Akida J, et al. Treatment of co-infection with bancroftian filariasis and onchocerciasis: a safety and efficacy study of albendazole with ivermectin compared to treatment of single infection with bancroftian filariasis. Filaria Journal. 2003 Nov 6;2(15):1-9.
20. Matamoros G, Sanchez A, Gabrie JA, Juarez M, Ceballos L, Escalada A, et al. Efficacy and Safety of Albendazole and High-Dose Ivermectin Coadministration in School-Aged Children Infected With Trichuris trichiura in Honduras: A Randomized Controlled Trial. Clinical Infectious Diseases. 2021 Oct 5;73(7):1203-10.
21. Ndyomugyenyi R, Kabatereine N, Olsen A, Magnussen P. Efficacy of ivermectin and albendazole alone and in combination for treatment of soil-transmitted helminths in pregnancy and adverse events: a randomized open label controlled intervention trial in Masindi district, western Uganda. American Journal of Tropical Medicine and Hygiene. 2008;79(6):856-63.
22. Edi C, Bjerum CM, Ouattara AF, Chhonker YS, Penali LK, Meite A, et al. Pharmacokinetics, safety, and efficacy of a single co-administered dose of diethylcarbamazine, albendazole and ivermectin in adults with and without Wuchereria bancrofti infection in Côte d’Ivoire. PLOS Neglected Tropical Diseases. 2019 May 20;13(5):1-15.
23. Garcia HH, Lescano AG, Lanchote VL, Pretell EJ, Gonzales I, Bustos JA, et al. Pharmacokinetics of combined treatment with praziquantel and albendazole in neurocysticercosis. British Journal of Clinical Pharmacology. 2011;21(1):77-84.
24. Garcia HH, Gonzales I, Lescano AG, Bustos JA, Zimic M, Escalante D, et al. Efficacy of combined antiparasitic therapy with praziquantel and albendazole for neurocysticercosis: a double-blind, randomized controlled trial. Lancet Infectious Diseases. 2014;14:687-95.
25. Kaur S, Singhi P, Singhi S, Khandelwal N. Combination Therapy With Albendazole and Praziquantel Versus Albendazole Alone in Children With Seizures and Single Lesion Neurocysticercosis. The Pediatric Infectious Disease Journal. 2009 May;28(5):403-6.
26. Lima RM, Ferreira MAD, de Jesus Ponte Carvalho, Teresa Maria, Fernandes BJD, Takayanagui OM, Garcia HH, et al. Albendazole-praziquantel interaction in healthy volunteers: kinetic disposition, metabolism and enantioselectivity. British Journal of Clinical Pharmacology. 2011;71(4):528-35.
27. Njenga SM, Ng’ang’a PM, Mwanje MT, Bendera FS, Bockarie MJ. A School-Based Cross-Sectional Survey of Adverse Events following Co-Administration of Albendazole and Praziquantel for Preventive Chemotherapy against Urogenital Schistosomiasis and Soil-Transmitted Helminthiasis in Kwale County, Kenya. PLOS One. 2014;9(2):1-5.
28. lds GR, King C, Hewlett J, Olveda R, Wu G, Ouma J, et al. Double-Blind Placebo-Controlled Study of Concurrent Administration of Albendazole and Praziquantel in Schoolchildren with Schistosomiasis and Geohelminths. The Journal of Infectious Diseases. 1999;179:996-1003.
29. Pengsaa K, Na-Bangchang K, Limkittikul K, Kabkaew K, Lapphra K, Sirivichayakul C, et al. Pharmacokinetic investigation of albendazole and praziquantel in Thai children infected with *Giardia intestinalis*. Annals of Tropical Medicine & Parasitology. 2004;98(4):349-57.
30. Namwanje H, Kabatereine NB, Olsen A. The acceptability and safety of praziquantel alone and in combination with mebendazole in the treatment of Schistosoma mansoni and soil-transmitted helminthiasis in children aged 1–4 years in Uganda. Parasitology. 2011 Feb 24;138:1586-92.
