# Supplementary material for: Safety of integrated preventive chemotherapy for neglected tropical diseases
Source: PLoS Negl Trop Dis. 2022 Sep 29;16(9):e0010700. doi: 10.1371/journal.pntd.0010700 (PMC9521808; doi:10.1371/journal.pntd.0010700)
Supplement: S2 Table — ALB, albendazole; AZI, azithromycin; DEC, diethylcarbamazine; IVM, ivermectin; LF, lymphatic filariasis; MDA, mass drug administration; MEB, mebendazole; MOH, Ministry of Health; NGO, Non-governmental organization; ONC, onchocerciasis; POS, powder for oral suspension; PZQ, praziquantel; SCH, schistosomiasis; STH, soil-transmitted helminths; TRA, trachoma. (DOCX) [file pntd.0010700.s004.docx]

| **Topic** | **Question** | **Responses** | **No. (%) MOH** | **No. (%) NGO** |
| --- | --- | --- | --- | --- |
| Co-Administration | Do you currently practice co-administration for MDA, with multiple medications given on the same day? | Yes | 8 (100.0) | 7 (100.0) |
|  |  | No | 0 (0.0) | 0 (0.0) |
|  | Are these drugs given together at the same time? | Yes | 8 (100.0) | 7 (100.0) |
|  |  | No | 0 (0.0) | 0 (0.0) |
|  | What diseases do you treat through co-administration during MDA? | LF + ONC | 2 (25.0) | 1 (14.3) |
|  |  | LF + ONC + STH | 0 (0.0) | 2 (28.6) |
|  |  | LF + ONC + SCH + STH | 0 (0.0) | 2 (28.6) |
|  |  | LF + SCH | 0 (0.0) | 2 (28.6) |
|  |  | LF + SCH + STH | 1 (12.5) | 1 (14.3) |
|  |  | LF + Yaws | 1 (12.5) | 0 (0.0) |
|  |  | Scabies + STH + TRA + Yaws | 1 (12.5) | 0 (0.0) |
|  |  | SCH + STH | 2 (25.0) | 1 (14.3) |
|  |  | STH + TRA | 1 (12.5) | 0 (0.0) |
|  | What drug combinations do you co-administer during MDA that are not currently recommended by WHO? | ALB + AZI | 2 (25.0) | 0 (0.0) |
|  |  | ALB + AZI + DEC + IVM | 1 (12.5) | 0 (0.0) |
|  |  | ALB + IVM + PZQ | 1 (12.5) | 4 (57.1) |
|  |  | IVM + MEB | 0 (0.0) | 1 (14.3) |
|  |  | IVM + PZQ | 0 (0.0) | 1 (14.3) |
| Tablet Crushing | When treating young children for lymphatic filariasis and soil-transmitted helminths during MDA, do you recommend crushing tablets? | Yes | 5 (62.5) | 2 (28.6) |
|  |  | No | 3 (37.5) | 5 (71.4) |
|  | If tablets are crushed, is water routinely given in conjunction? | Yes | 4 (50.0) | 4 (57.1) |
|  |  | No | 4 (50.0) | 1 (14.3) |
|  | What drugs do you recommend crushing during MDA? | Albendazole | 4 (50.0) | 1 (14.3) |
|  |  | Azithromycin | 3 (37.5) | 0 (0.0) |
|  |  | Mebendazole | 0 (0.0) | 1 (14.3) |
|  |  | Praziquantel | 1 (12.5) | 2 (28.6) |
| Parent Involvement | Are parents allowed to give medicine to their children during MDA? | Yes | 8 (100.0) | 3 (42.9) |
|  |  | No | 0 (0.0) | 3 (42.9) |
|  | If parents are allowed to give children drugs during MDA, how is treatment observed? | Observed by distribution team | 8 (100.0) | 4 (57.1) |
|  |  | Not observed – parents allowed to take tablets home | 1 (12.5) | 1 (14.3) |
| Child Refusal | If a child refuses to take tablets during MDA, what steps are taken? | Encouraging child | 4 (50.0) | 3 (42.9) |
|  |  | Not forcing drug and marking as refusal | 6 (75.0) | 3 (42.9) |
|  |  | Bringing child back | 1 (12.5) | 3 (42.9) |
|  |  | POS offered (for AZI) | 2 (25.0) | 0 (0.0) |
|  |  | Parent / community member calming child | 1 (12.5) | 0 (0.0) |
| Neglected Tropical Disease Integration | If it were safe to give all appropriate NTD drugs during a single day of MDA, would you find that useful? | Yes | 5 (62.5) | 6 (85.7) |
|  |  | No | 2 (25.0) | 1 (14.3) |
|  | What drugs would you like to be able to give together during MDA that you currently don’t because their co-administration is not recommended by WHO? | ALB + AZI + DEC + IVM | 1 (12.5) | 0 (0.0) |
|  |  | ALB + AZI + IVM | 2 (25.0) | 1 (14.3) |
|  |  | ALB + AZI + IVM + PZQ | 0 (0.0) | 1 (14.3) |
|  |  | ALB + IVM + PZQ | 1 (12.5) | 0 (0.0) |
|  |  | IVM + PZQ | 1 (12.5) | 1 (14.3) |
